# Bilateral Superior Cervical Sympathectomy Activates Signal Transducer and Activator of Transcription 3 Signal to Alleviate Myocardial Ischemia-Reperfusion Injury

**DOI:** 10.3389/fcvm.2022.807298

**Published:** 2022-04-01

**Authors:** Lixia Li, Jiahong Gao, Lin Gao, Le Li, Hongfei Zhang, Wei Zhao, Shiyuan Xu

**Affiliations:** Department of Anesthesiology, Zhujiang Hospital, Southern Medical University, Guangzhou, China

**Keywords:** superior cervical sympathectomy, STAT3, oxidative stress, apoptosis, inflammation, myocardial ischemia-reperfusion injury

## Abstract

**Background:**

There is growing evidence about the effect of bilateral superior cervical sympathectomy on myocardial ischemia-reperfusion (I/R) injury. Studies have increasingly found that the signal transducer and activator of transcription 3 (STAT3) plays a protective role in myocardial I/R injury. However, the precise mechanism is unknown. The present study explored the bilateral superior cervical sympathectomy’s effect and potential mechanism in mice myocardial I/R injury.

**Methods:**

The left heart I/R injury model was created by ligating the anterior descending branch of the coronary artery for 30 min followed by reperfusion. Bilateral superior cervical sympathectomy was performed before myocardial I/R injury. To evaluate the effect of bilateral superior cervical sympathectomy on the myocardium, we examined the myocardial infarct size and cardiac function. Then, myocardial apoptosis, inflammation, and oxidative stress were detected on the myocardium. Furthermore, the expression of STAT3 signal in myocardial tissue was measured by western blotting. To further examine the cardioprotective effect of STAT3 after bilateral superior cervical sympathectomy, the STAT3 inhibitor (static) was utilized to inhibit the phosphorylation of STAT3.

**Results:**

The results showed that the myocardial I/R injury decreased and the cardiac function recovered in the myocardial I/R injury after cervical sympathectomy. Meanwhile, cervical sympathectomy reduced the myocardial distribution of the sympathetic marker tyrosine hydroxylase (TH) and systemic sympathetic tone. And levels of oxidative stress, inflammatory markers, and apoptosis were reduced in myocardial tissue. We also found that the STAT3 signal was activated in myocardial tissue after cervical sympathectomy. STAT3 inhibitor can partially reverse the myocardial protective effect of cervical sympathectomy.

**Conclusion:**

Bilateral superior cervical sympathectomy significantly alleviated myocardial I/R injury in mice. And activation of the STAT3 signal may play an essential role in this.

## Introduction

Acute myocardial infarction (AMI) is a common health problem worldwide (1). The primary management strategy for AMI is to restore coronary blood flow promptly, and the early revascularization rate in patients with AMI increases year by year (2). However, the return of blood flow through reperfusion may inevitably result in additional damage to cardiomyocytes, called ischemia reperfusion (I/R) injury (3). Many experimental and clinical evidence shows that acute myocardial ischemia can induce intense activation of sympathetic nervous system (SNS), remaining elevated long-term and potentially irreversible (4, 5). Activation of the sympathetic nervous system triggers subsequent arrhythmias and leads to direct myocardial damage, including affecting the extent of infarct size (6, 7). Reperfusion of ischemic myocardium produces a large number of reactive oxygen species reactive oxygen species (ROS). Previous studies have shown that ROS stimulation of cardiac afferent vasopressor responses is enhanced by vagotomy and abolished by sympathectomy (8). It is worth considering that ROS-mediated damage is closely related to sympathetic nerves. This study aimed to explore a new pathway for alleviating myocardial I/R injury.

Several approaches have been developed that aim at the local interference with the sympathetic innervation of the heart, demonstrating that myocardial sympathetic denervation alleviates the harmful progression of many cardiovascular diseases. Cardiac sympathetic denervation (CSD) is a clinically effective strategy to treat patients with malignant ventricular arrhythmias (9, 10). In the rat model of myocardial infarction, bilateral stellate ganglion resection effectively reduced left ventricular remodeling and myocardial cell apoptosis and improved cardiac function (11). In addition, studies have demonstrated that left stellectomy increased survival of the myocarditis rats while showing antiarrhythmic effects with reduced inflammation (12). Similarly, inhibition of the augmented cardiac sympathetic afferent reflex is beneficial for preventing ventricular arrhythmias caused by AMI (13).

However, the effects and mechanism of ganglionectomy on experimental myocardial I/R injury models are not fully understood. All sympathetic nerves follow the blood vessels to the target organs, a phenomenon is known as neurovascular congruence. The Superior cervical ganglion (SCG) is located at the bifurcation of the internal and external carotid arteries and projects laterally along these arteries to the head and neck (14). Webb et al. proposed in the clinical report that in the hyperacute stage of human myocardial infarction, cardiovascular changes have a great relationship with the infarction site, and anterior wall infarction is mostly associated with sympathetic hyperactivity (15). Part of the SCG nerve projects toward the anterior wall of the heart, which overlaps with the vegetative region of the left anterior descending artery (LAD). SCG plays a vital role in different cardiovascular diseases (16, 17). In animal models, previous studies have demonstrated that myocardial infarction increases SCG neuronal activity by affecting ion channel opening and the amplitude of action potential (18). Furthermore, in cell studies, the co-culture of neonatal rat SCG neurons with neonatal rat cardiomyocytes for 24 h induces Ca^2+^ processing and release from cardiomyocytes and does not occur spontaneously in neurons grown alone (19). SCG has a significant effect on the physiological activity of the myocardium. To our best knowledge, this is the first experimental study reporting on the impact of bilateral SCG and related mechanisms in this I/R injury mice model. The transcription factor, signal transducer and activator of transcription 3 (STAT3) have been implicated in protecting the heart from acute ischemic injury. The protein level and activation status of STAT3 are dynamic, as is its subcellular distribution. STAT3 has 14 highly conserved cysteine residues, nine of which are reported to be sensitive to redox activity and are closely related to the activation of tyrosine residue phosphorylation (20–22).

Furthermore, the transcription activation domain (TAD) contains a second conserved phosphor-amino acid residue at the C-terminal, phospho-serine (Ser727), which is critical for maximum transcriptional activation of STAT3 (23). Phosphorylation of STAT3 leads to its dimerization and subsequent translocation into the nucleus to interact with regulatory elements for gene expression (24, 25). Many studies have reported the protective effect of phosphorylation of Tyr705 on myocardial I/R injury. Calycosin isoflavone-7-O-β-D-glucoside (CG) is one of the main components of astragalus membranaceus (AR) with anti-inflammatory and antioxidant activities. It has been reported that CG preconditioning activates JAK2/STAT3 signaling pathway by up-regulating the expression of IL-10, which helps protect the myocardium from I/R injury (26). It was also reported that IL-10 increases secreted galectin-3 and osteopontin expression *via* phosphorylating the Tyr705 residue of STAT3, which repairs the heart after myocardial infarction (27). The protection conferred by STAT3 is related to the regulation of myocardial processes such as anti-cardiomyocyte apoptosis anti-inflammatory and anti-oxidative stress (20, 21).

In the current study, we tested the hypothesis that bilateral SCG removal might be an effective strategy for the partial denervating of the heart *via* lowering the expression of cardiac sympathetic neurohormones and subsequently attenuating the inflammatory response, oxidative stress, and apoptosis of myocardial cells. Furthermore, we aimed to investigate that bilateral superior cervical sympathectomy exerts cardioprotective effects in myocardial I/R injury mice by activating the STAT3 signaling.

## Materials and Methods

### Animals

A total of 120 healthy specific pathogen-free (SPF) C57BL/6 wild-type (*wt*) mice at 8–10 weeks were purchased from Guangdong Animal Center (Guangzhou, China). After 4–7 days of quarantine, all mice were fed in the Laboratory Animal Center with a regular diet and sterile filtered water every day under the conditions of a 12/12 h light/dark cycle, with temperatures ranging from 18 to 24°C and 60–65% humidity. Adapt to the feeding environment for at least 1 week before the formal experiment. The study was approved by the Institutional Animal Care and Use Committee of Zhujiang Hospital of Southern Medical University (Item no. LAEC-2020-099).

### Myocardial Ischemia-Reperfusion in Mice

Ischemia-reperfusion was induced on 11–13-week-old male mice. Subcutaneous injection of buprenorphine (0.08 mg/kg) was used for preoperative and postoperative analgesia. Mice were anesthetized *via* intraperitoneal injection of 1% pentobarbital sodium (40 mg/kg, i.p.) and fixed in the supine position. The mice were intubated, and ventilator parameters were set as respiratory rate 110 times/min, tidal volume 0.8 ml, airway peak pressure 35–45 cmH2O (Havard). The fourth intercostal space over the left chest of the mouse was exposed. The left anterior descending coronary artery (LAD) was found and ligated with 8–0 ophthalmic suture. Myocardial ischemia was confirmed when the left anterior wall turned pale and the ECG showed ST-segment elevation and high-amplitude T wave. After 30 min, the ligature was loosened for 24h of reperfusion. Only the thorax and pericardium were opened in the sham operation without ligating the left anterior descending branch.

### Cervical Sympathetic Ganglionectomy

Mice were anesthetized with 1.5% isoflurane and a vertical incision was made in the neck. The glands and muscles were obtusely separated by microscopic tweezers. Laterally to the left sternocleidomastoid muscle has a pulsing common carotid artery, which follows in cranial direction to find the carotid bifurcation (internal and external carotid). The SCG is located behind the carotid bifurcation. With preganglionic and postganglionic branches, the SCG is located behind the carotid artery bifurcation. The SCG was carefully separated from the sympathetic nerve chain and the SCG tissue was collected. The contralateral SCG was isolated in the same manner.

### Animal and Experiment Experimental Groups

120 mice were randomly separated into five equal groups (*n* = 6): the Con group, the SCGx group, the IR group, the SCGx + IR group and the Stattic group, the success rate of the modeling was 80%. The number of successful models was 96. 24 mice were used for Evans blue-TTC double staining without collecting heart and blood samples. 36 mice were used for immunohistochemical experiments. 36 mice were used for PCR, Western Blot and enzyme-linked immunosorbent assay (ELISA) assay. In the CON group, only the ribs and pericardium were opened without ligation, and SCG was separated but not broken. In the SCGx group, bilateral ganglionectomy and only the ribs and pericardium were opened without ligation. In the IR group, LAD ligation was ligation for 30 min and reperfusion for 24h, SCG was separated but not broken. In the SCGx + IR group, bilateral ganglionectomy (SCGx) followed by LAD Ligation for 30 min and reperfusion for 24h. In order to avoid the acute inflammatory reaction period, IRI surgery was performed under pentobarbital and buprenorphine anesthesia 3 days later. In the Stattic group, bilateral ganglionectomy (SCGx) followed by LAD Ligation for 30 min and reperfusion for 24h, stattic (Selleck, 20 mg/kg, i.p.) was administered 40 min before ischemia. STAT3 inhibitor, stattic (Selleck, 20 mg/kg, i.p.), a small non-peptide molecule that potently inhibits STAT3 activation and nuclear translocation, was administered 40 min before ischemia in the Stattic group (28). 24 h after surgery, blood samples were collected from the retrobulbar venous plexus for ELISA test, and heart tissue was carefully removed and stored at −80°C.

### Echocardiography

Echocardiographic monitoring was carried out before surgery and at the end of the experiment before the tissue was harvested. M-mode echocardiography was obtained using a small-animal ultrasound probe (model Veno2100) on the long axis of the parastolic left ventricle. LVIDs (left ventricular internal dimension systole) and LVIDD (left ventricular internal diastolic diameter) were recorded. Mice were anesthetized with 1.5% isoflurane, and the heart rate, respiration rate, and electrocardiogram were monitored. Myocardial contractility was assessed by the ejection fraction (EF) and fractional shortening (FS). All parameters were averaged over five cardiac cycles for analysis.

### Enzyme-Linked Immunosorbent Assay

The blood samples (1 ml) were collected from the retrobulbar venous plexus. The serum creatine kinase (CK-MB) and lactate dehydrogenase (LDH) levels were measured according to kits instructions (enzyme-linked immunosorbent assay (ELISA), Nanjing Jiancheng Bioengineering Institute, Jiangsu, China). According to the manufacturer’s instructions, the plasma’s NE (norepinephrine) levels were measured by ELISA assay kits (MEIMIAN, China).

### Evans Blue-Triphenyltetrazolium Chloride Double Staining Methods

Mice were anesthetized with 1% pentobarbital sodium after 24 h of reperfusion. The LAD was blocked again, and Evens blue dye (2% w/v, Sigma-Aldrich) was injected into the ascending aorta to identify area-at-risk (AAR) and non-ischemic normal areas. The hearts were then frozen and sliced into 1 mm thick pieces. Slices were stained by a 2% (w/v) triphenyltetrazolium chloride (TTC; Sigma Aldrich) for 15–20 min at 37°C to identify ischemic tissue and infarction area. The hearts were immersed in a 4% aqueous solution of formaldehyde for 24 h. The infarct size was digitally measured using ImageJ analysis software (Image J, Version 1.47, National Institutes of Health, Bethesda, MD, United States). The infarct area was expressed as a percentage of the AAR.

### Hematoxylin–Eosin Staining

Mice hearts were paraffin-embedded and cut into 4 μm slices. The sections were stained with hematoxylin for 3 min, washed with tap water, stained with eosin for the 30 s, and dehydrated in graded ethanol. Five fields were randomly selected to observe the morphological characteristics of infracted tissues under the light microscope.

### Immunohistochemical Staining

The infarct border area of the heart was selected. Myocardium embedded in paraffin was sliced into 5 μm slices, followed by dehydration in graded alcohol and deparaffinization in dimethylbenzene. To achieve adequate antigen retrieval, the sections were immersed in a citric acid buffer solution and were repeatedly heated in a microwave oven 3 times (7 min/time). The sections were incubated in 3% H_2_O_2_ for 10 min to block the endogenous peroxidase and added with goat serum in drops for 30 min to block non-specific antigens. The TH antibody (1:500 diluted in PBS, Abcam) was added and incubated overnight in a refrigerator at 4°C. The next day, added dropwise with the secondary antibody and added with a streptavidin-peroxidase solution for 10 min. Finally, diaminobenzidine (DAB) was used to develop color and hematoxylin was used to reverse stain the nucleus.

### TdT-Mediated dUTP-Biotin Nick End-Labeling Staining

After 4% paraformaldehyde was perfused from the aorta, myocardial tissue (the border risk area) was soaked in 4% paraformaldehyde solution for 24 h. Dehydrated with 20% sucrose, embedded the tissue with OCT, then the myocardium was cut into 8 μm thick at a cryostat. Cell apoptosis was detected by the TdT-mediated dUTP-biotin nick end-labeling (TUNEL) kit (KEYGEN, Nanjing, China). PBS was used as negative control instead of a primary antibody. Finally, stain the nucleus and sealed pieces with 4-6-diamino-2-phenylindole (DAPI)(Abcam). Five views were randomly selected to calculate TUNEL positive nucleus under the fluorescence microscope. The apoptosis rate was expressed as the percentage of apoptotic nucleus relative to the total number of DAPI-stain nucleus.

### Detection of Malondialdehyde Content and Superoxide Dismutase Activity

In total, 20 mg of cardiac tissue was weighed and added to PBS (PH7.4), which was quickly ground into 10% tissue homogenate in the mortar. The supernatant was centrifuged and collected to detect changes in superoxide dismutase (SOD) (Nanjing Jiancheng, A001-1-1) and malondialdehyde (MDA) (Nanjing Jiancheng, A003-1-1) levels. The absorbance of each index was measured using a microplate reader.

### Real-Time Polymerase Chain Reaction Measurements

The real-time polymerase chain reaction was performed to detect the expression levels of IL6, IL-1β, TNF-α and IL-10 by Bio-Rad CFX96 (Bio-Rad, Hercules, CA, United States) according to the manufacturer’s instructions. We used AG RNAex Pro Reagent to extract RNA from myocardial tissue. 5 × Evo M-MLVRT Master Mix was used for cDNA synthesis, and 2X SYBR^®^ Green Pro Taq HS Premix was used for primer amplification (Accurate biology, China). The amplification reaction conditions were 37°C for 15 min, 85°C for 5 s, and 4°C for infinity. Relative gene expression was calculated using the 2-ΔΔCT method. The primer sequences used in this study are shown below. Sense GCAACTGTTCCTGAACTCAACT and anti-sense ATCTTTTGGGGTCCGTCAACT for IL-1β, sense GCTCTTACTGACTGGCATGAG and anti-sense CGCAGCTCTAGGAGCATGTG for IL-10; sense CGAGTGACAAGCCTGTAGCC and anti-sense GGTGAGGAGCACGTAGTCG for TNF-α, sense TAGTCCTTCCTACCCCAATTTCC and anti-sense TTGGTCCTTAGCCACTCCTTC for IL-6, sense GGTTGTCTCCTGCGACTTCA and anti-sense TGGTCCAGGGTTTCTTACTCC for GAPDH.

### Western Blotting Assay

The cryopreserved myocardial tissue was treated by RIPA lysis buffer containing protease and phosphatase inhibitors for protein extraction. 20 μg protein samples were loaded on 10% SDS-PAGE gels, transferred to PVDF membranes, and blocked in 5% skim milk powder for 1.5 h. PVDF membranes were incubated with anti-STAT3 (Abcam, ab68153), anti-p-STAT3 (Abcam, ab76315), anti-Bcl-2 (CST, 3498S), anti-Bax (CST, 14796S), anti-TH (Abcam, ab137869), anti-GAPDH (Proteintech, 6004-1-2 g), and anti-IL-10 (R&D, AF519-SP) at 4°C overnight. After washing with *Tris*-buffered Saline with 0.05% Tween-20 (TBST), the membranes were incubated with secondary antibody for 1 h, followed by rinsing again with TBST. Immunoreactive bands were exposed using the enhanced chemiluminescence (ECL) reagent in a dark room. The relative expression of the protein was analyzed using ImageJ analysis software.

### Data Analysis

The data are expressed as the mean ± standard deviation (SD). Statistical analysis was performed with SPSS20.0 statistical software. Data distribution was assessed by Shapiro–Wilk test for normality, and equal variance was assessed by the Brown-Forsythe test. Analysis of variance followed by Bonferroni test (normally distributed data set). If the normality or equal variance test fails, Kruskal–Wallis rank univariate analysis of variance (ANOVA) is used, followed by Dunn’s multiple comparison test. It was performed with an unpaired *t*-test when comparing two different groups. Statistical significance was the analysis of variance *p* < 0.05.

## Results

### Sympathetic Denervation Improved Cardiac Function and Reduced Infarct Size in Ischemia-Reperfusion Injury Mice

**FIGURE 1:**
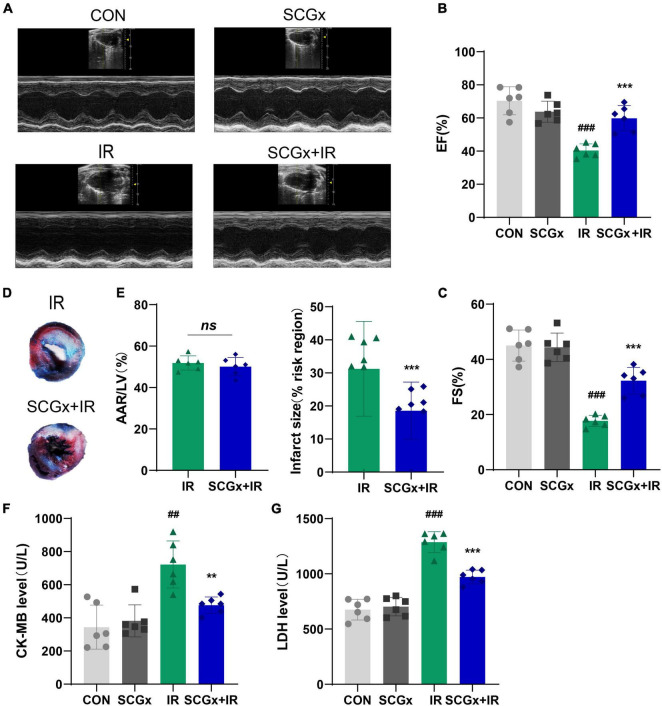
Sympathetic denervation improves cardiac function in mice with myocardial I/R. **(A–C)** Representative M-mode echocardiography images. LVEF, left ventricular ejection fraction. LVFS, left ventricular fractional shortening. **(D)** Representative photographs of Evans blue and 2,3,5-triphenyltetrazolium chloride double-stained heart sections. **(E)** Quantitative analysis of the proportion of myocardial infarction area. **(F,G)** Detection of creatine kinase-MB (CK-MB) and lactate dehydrogenase (LDH) in mice serum. *n* = 6, 0.001 < **P < 0.01 and ***P < 0.001 compared with the I/R group. 0.001 < ^##^*P* < 0.01 and ^###^*P* < 0.001 compared with the CON group.

Twele-week-old C57/BL6J mice were randomly assigned to the control and SCGx groups before ligation of the left anterior descending branch. To verify that the removed structure indeed contained the ganglionic sympathetic neurons, we performed immunofluorescent staining with an antibody directed against TH (Supplementary Figure 2). The treated mice were then randomly divided into the control and I/R groups. As shown in Figures 1A–C, cardiac function was impaired after myocardial reperfusion injury, manifested as decreased LVEF and LVFS (*P* < 0.001 vs. CON). Removal of bilateral superior cervical sympathetic nerves significantly improved cardiac function in I/R injury mice, as evidenced by increased left ventricular ejection fraction and the rate of short-axis shortening (*P* < 0.001 vs. I/R). However, bilateral superior cervical sympathectomy did not affect LVEF and LVFS in normal mice (Supplementary Figure 1). Furthermore, in the case of the same risk area, larger infarct size was observed in heart tissues of normal mice undergoing I/R injury, but the size was decreased in mice with pre-bilateral superior cervical sympathectomy experiencing myocardial I/R injury. The infarct area of the SCGx + IR group decreased significantly compared with the I/R group (*P* < 0.01 vs. I/R, Figures 1D,E). Moreover, the enzyme-linked immunosorbent assay (ELISA) was used to determine the serum levels of cardiac injury markers CK-MB and LDH (Figures 1F,G). We found that mice undergoing I/R injury presented elevated CK-MB and LDH relative to sham-operated mice (*P* < 0.01 vs. CON). Pretreatment of sympathetic denervation, I/R-injured mice showed reduced serum CK-MB and LDH levels (*P* < 0.01 vs. I/R).

### Removal of Bilateral Superior Cervical Ganglion Effectively Reduces Sympathetic Innervation of the Anterior Myocardial Wall

**FIGURE 2:**
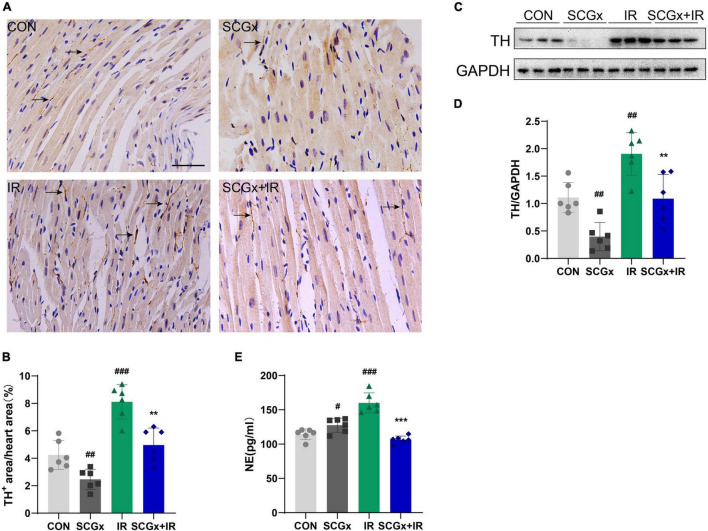
Sympathetic denervation reduces the sympathetic excitability of myocardium. **(A,B)** Quantification of intramyocardial sympathetic nerve by immunohistochemistry staining with an antibody directed against TH. Arrows indicate the immunostaining neurons. scale bar: 100 μm. (*n* = 6) **(C,D)** The expression of TH protein in heart tissue was examined using western blotting analyses. (*n* = 6) **(E)** Detection of Norepinephrine (NE) in mice serum. *n* = 6, 0.001 < **P < 0.01, and ***P < 0.001 compared with the I/R group. ^#^*P* < 0.05, 0.001, ^##^*P* < 0.01, and ^###^*P* < 0.001 compared with the CON group.

Studies have shown that the imbalance of cardiac autonomic nerve, that is, decreased vagal activity and excessive sympathetic activity, is related to the pathogenesis of myocardial I/R injury (29, 30). Activation of the sympathetic nervous system is a crucial initiator of subsequent inflammatory responses and is associated with the extent of myocardial infarction. SNS activation is related to the release of monocytes macrophages, the expression of various cytokines and the generation of B cell antibodies (31–34). To determine the effect of local cardiac denervation on cardiac nerve germination and sympathetic overactivation, we performed immunohistochemical staining of the left ventricle, where TH is a rate-limiting enzyme in catecholamine and synthesis sympathetic marker (Figure 2A). We also measured serum norepinephrine, a neurohormone that can reflect SNS activity. We then used TH staining to detect sympathetic endings on the anterior wall of the left ventricle (the ischemic area). Bilateral SCGx significantly reduced the number of Th^+^ neurons in the anterior myocardial wall of normal mice (*P* < 0.01 vs. CON Figure 2B). Cardiac NE overflow is often used as an indirect measure of sympathetic activity. However, the serum NE level did not decrease (Figure 2E). Studies have shown that disruption of norepinephrine in infarct and periinfarct myocardium is accompanied by an abnormal increase in plasma norepinephrine (35, 36). We hypothesized that systemic sympathetic excitability could be compensated by local sympathetic denervation in the myocardium. In our study, serum NE levels and Th^+^ neurons were significantly increased in the I/R group. Interestingly, unlike normal mice, pre-bilateral superior cervical sympathectomy can reduce the increase of local and systemic myocardial sympathetic excitability after I/R injury, as evidenced by decreased NE level of serum and TH levels of the myocardial border zone in the SCGx + IR group (*P* < 0.01 vs. IR Figures 2B,E). Moreover, western blotting was used to detect TH protein in the border zone of the myocardium, and the results were consistent with the above (Figures 2C,D).

### Sympathetic Denervation Inhibited Apoptosis and Oxidative Stress in Myocardial Ischemia-Reperfusion Mice

**FIGURE 3:**
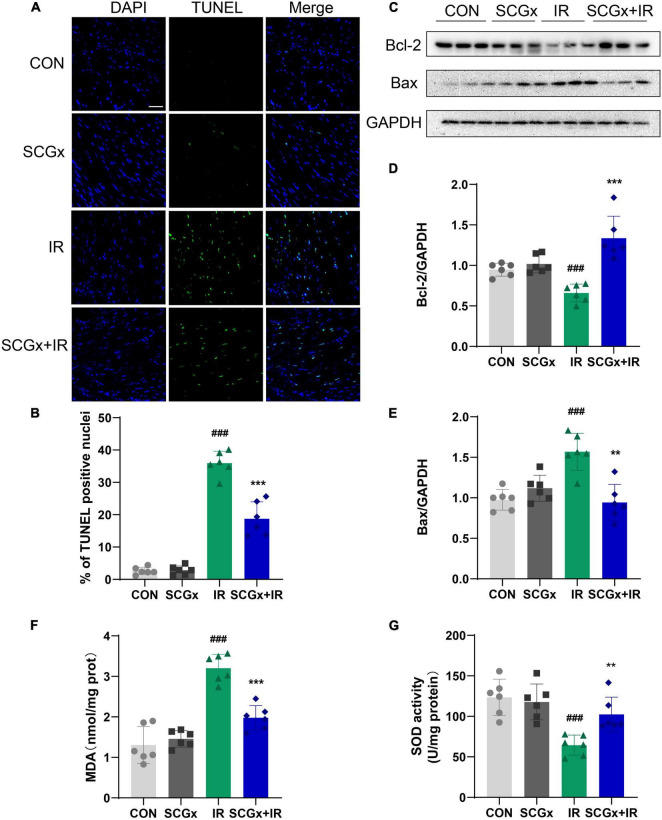
The level of apoptotic cells and oxidative stress decreased upon sympathetic denervation. **(A)** Cell apoptosis was detected *via* TUNEL. scale bar: 50 μm. **(B)** Percentage of TUNEL positive nuclei in each group. **(C–E)** The expression of Bcl-2 and Bax in heart tissue. **(F,G)** Detection of SOD and MDA in heart tissue. *n* = 6, 0.001 < **P < 0.01 and ***P < 0.001 compared with the I/R group. ^###^*P* < 0.001 compared with the CON group.

Apoptosis is the critical event of myocardial I/R injury. Recent studies have demonstrated that in areas where necrotic cells are not present to any significant amount, a good correlation is found between the TUNEL test and other more sophisticated methods (37). As shown in Figures 3A,B, we performed TUNEL staining and found I/R induced prominent apoptosis in normal mice heart tissues (*P* < 0.001 vs. CON). SCG removal led to a significant decrease of these apoptotic cells (*P* < 0.01 vs. I/R). Pro-apoptotic protein Bax can form a heterodimer with anti-apoptotic protein Bcl-2 and inhibit Bcl-2. It was found that the ratio of Bax to Bcl-2 protein was the key factor to determine the intensity of apoptosis inhibition (38). The expression levels of Bax and Bcl-2 in heart tissue were detected by Western blot (Figures 3C–E). The results indicated that the protein expression of Bcl-2 was remarkably declined in the I/R group than those in the control group (*P* < 0.001 vs. CON), while Bax was significantly increased in the I/R group than those in the control group (*P* < 0.001 vs. CON). Moreover, the expression of the Bcl-2 protein was remarkably increased, while the expression of Bax protein was significantly decreased in the SCGx + IR group (*P* < 0.01 vs. IR). These results suggested that sympathetic denervation had an anti-apoptosis effect on myocardial cells in I/R injury mice.

The change of SOD activity in tissues can indirectly reflect the ability to scavenge oxygen free radicals. The content of MDA can indirectly reflect the degree of lipid peroxidation and the degree of cell damage. Myocardial I/R injury induced significant oxidative stress (as evidenced by increased MDA level and decreased SOD level) in mice, but it was significantly reduced in mice subjected to sympathectomy. As shown in Figures 3F,G, the MDA content declined (*P* < 0.001), the SOD activity was greatly enhanced in the SCGx + IR group (*P* < 0.01 vs. I/R).

### Attenuated Ischemia-Reperfusion-Induced Myocardial Injury and Inflammatory Cell Infiltration Upon Sympathetic Denervation

**FIGURE 4:**
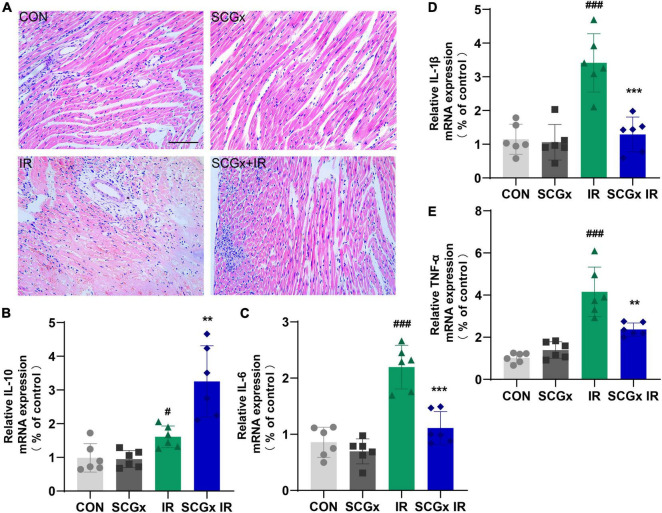
Effect of sympathetic denervation on relative expression levels of inflammatory cytokines and pathological changes in heart tissue of myocardial I/R mice. **(A)** H-E staining. scale bar: 100 μm **(B–E)** Determination of inflammation-related mRNAs by PCR in total RNA prepared from myocardium. interleukin-10 (IL-10) and interleukin-6 (IL-6), Interleukin-1β (IL-1β), tumor necrosis factor alpha (TNF-α). *n* = 6, 0.001 < **P < 0.01 and ***P < 0.001 compared with the I/R group. ^#^*P* < 0.05 and ^###^*P* < 0.001 compared with the CON group.

As shown in Figure 4A, the myocardial cells were evenly stained. The cardiac fibers were observed to arrange neatly. The cells are well-arranged and morphology integral in the control group. In heart tissues of mice undergoing I/R injury, the myocardial cells were chaotic, with uneven cytoplasm staining, accompanied by vacuoles, myocardial rupture, and inflammatory cell infiltration. These symptoms were relieved in the sympathetic denervation model mice.

As expected, myocardial I/R injury induced a prominent and long-lasting myocardial infiltration of inflammatory cells. We sought to determine whether the myocardial protective effect of the sympathetic denervation is related to the regulation of inflammation. In the case of sympathetic innervation, Real-time PCR results demonstrated that the expression levels of IL-1β, IL-6 and TNF-α mRNA were significantly increased in the I/R group compared with those in the CON group. However, the IL-1β, IL-6 and TNF-α mRNA levels in the SCGx + IR group were greatly relieved with sympathetic denervation. Compared with the IR group, the IL-10 level was significantly elevated in the SCGx + IR group (Figures 4B–E). Both IL-10 and IL-6 induce activation of STAT3, but IL-6 induces proliferation and the production of inflammatory cytokines that promote tumor growth and is also considered to be a strong driver of many chronic inflammatory diseases (39). IL-10 binding to IL-10R activates the JAK/STAT3 cascade, where phosphorylated STAT3 homodimers translocate to the nucleus within seconds to activate the expression of target genes (40). IL-10 signaling induces a solid anti-inflammatory response (41).

### Sympathetic Denervation Activates the Signal Transducer and Activator of Transcription 3 Signal in Mice With Myocardial Ischemia-Reperfusion Injury

**FIGURE 5:**
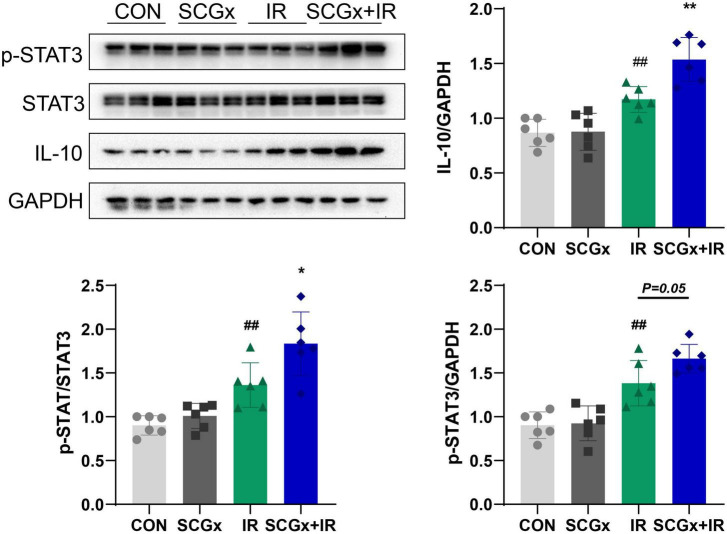
Sympathetic denervation activates the *p*-STAT3 signal in mice with myocardial I/R injury. The expression of IL-10 and *p*-STAT3 in heart tissue. *n* = 6, *P < 0.05 and 0.001 < **P < 0.01 compared with the I/R group. 0.001 < ^##^*P* < 0.01 compared with the CON group.

Tyrosine phosphorylation at Tyr705 of latent STAT3 is regulated by H_2_O_2_ (31, 42). As shown in Figure 5, in the case of sympathetic innervation, western blotting showed that anti-inflammatory factor IL-10 and p-STAT3 levels were significantly increased after I/R injury at 24 h (*P* < 0.01 vs. CON). Interestingly, with sympathetic denervation, I/R injury mice with SCG removal further increased relative IL-10 and p-STAT3 levels compared with those in I/R (*P* < 0.05, Figure 5). The Removal of SCG pretreatment significantly increased the protein expression levels of IL-10 and STAT3 in myocardial I/R injury mice. STAT3-mediated cardiac protection is achieved at least in part by enhancing the transcriptional activity of STAT3 by phosphorylation of Tyr705. The above results indicated that the protective effect of sympathetic denervation on myocardial I/R in mice might be related to the activation of the STAT3 signal.

### Stattic Could Partially Blunt the Protective Effect of Sympathetic Denervation on the Myocardial Ischemia-Reperfusion Injury

**FIGURE 6:**
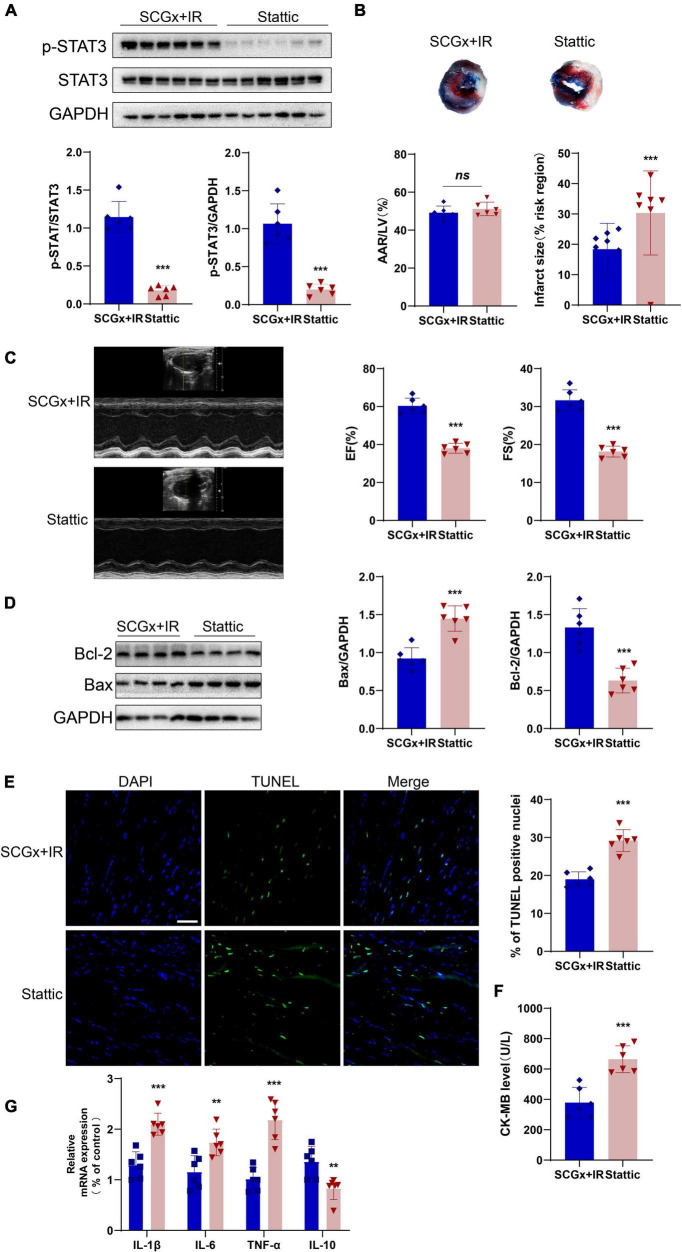
Stattic partially attenuated the protective effect of sympathetic denervation on the myocardial ischemia-reperfusion injury. **(A)** The expression of *p*-STAT3 protein was significantly inhibited by stattic (20mg/kg, *ip*). **(B)** Representative photographs of Evans blue and 2,3,5-triphenyltetrazolium chloride double-stained heart sections. **(C)** Representative M-mode echocardiography images. left ventricular ejection fraction (LVEF), left ventricular fractional shortening (LVFS). **(D)** The expression of Bcl-2 protein was decreased after *p*-STAT3 protein was inhibited, and Bax protein was increased. **(E)** Representative fluorescent images and percentage of TUNEL positive nuclei in each group. **(F)** Quantitative analysis of the proportion of myocardial infarction area. Detection of creatine kinase-MB (CK-MB) in mice serum. **(G)** Determination of inflammation-related mRNAs by PCR in total RNA prepared from the myocardium. interleukin-10 (IL-10) and interleukin-6 (IL-6), Interleukin-1β (IL-1β), tumor necrosis factor alpha (TNF-α). *n* = 6, 0.001 < **P < 0.01 and ***P < 0.001 compared with the SCGx + IR group.

The STAT3-specific inhibitor, stattic, significantly reduced p-STAT3 in myocardial tissues (*P* < 0.001 vs. SCGx + IR, Figure 6A). As shown in Figure 6B, the size of myocardial infarction in the stattic group was significantly higher than that in the SCGx + IR group. Figure 6C exhibited that cardiac function was impaired compared with the SCGx + IR group after inhibiting myocardial STAT3 phosphorylation, manifested as decreased LVEF and LVFS. Except for that, inhibition of STAT3 phosphorylation elevated the apoptosis rate of the myocardium. Western blotting demonstrated that the protein expression of Bcl-2 was remarkably decreased, while Bax was significantly increased in the stattic group (*P* < 0.001 vs. SCGx + IR, Figure 6D). A similar pattern was observed in the number of myocardial apoptotic cells. Stattic preconditioning resulted in a significant increase in the numbers of TUNEL-positive nucleus (*P* < 0.01 vs. SCGx + IR Figure 6E). As shown in Figure 6F, we detected the level of CK-MB, suggesting that it was significantly increased in the Stattic group (*P* < 0.001 vs. SCGx + IR, Figure 6F). We also measured the levels of inflammatory factors in the myocardium, and the results showed that anti-inflammatory factors decreased and pro-inflammatory factors increased in the Stattic group. (*P* < 0.01 vs. SCGx + IR, Figure 6G). The protective effect of SCG removal on the myocardial ischemia-reperfusion injury was partially blunted by the STAT3 inhibitor Stattic, as evidenced by decreased levels of the anti-apoptotic protein Bcl-2, increased number of apoptotic cells and the levels of cardiac injury markers enhanced inflammatory response. In summary, these data suggest that sympathetic denervation activates the STAT3 signal to protect the myocardial ischemia-reperfusion injury.

## Discussion

Sympathetic nervous system plays a vital role in the occurrence and development of myocardial I/R injury (7). It plays an essential role in regulating various physiological functions such as cardiovascular, metabolism, inflammation and immunity (43, 44). Previous studies have shown that activation of the sympathetic nervous system is a pivotal contributor to inflammatory reactions and associated with the extent of infarct size. The primary neurotransmitters in the SNS are norepinephrine (NE), adenosine triphosphate (ATP) and neuropeptide Y (NPY) (34). Local sympathetic nerves mainly secrete NE in peripheral organs, involved in the direct regulation of immune cells expressing adrenergic receptors (45). Studies have reported that NE rapidly induces IL-10 secretion from innate cells in response to multiple toll-like receptor (TLR) signals, and this effect is mediated by β2 adrenergic receptor (ADRB2) (46). When myocardial I/R injury occurs, the intense inflammatory response is accompanied by NE-mediated anti-inflammatory mechanism. Compared with the CON group, the I/R group showed higher sympathetic excitability and detected increased IL-10 levels in our study. This can be considered a passive increase of anti-inflammatory factors in response to oxidative stress, insufficient to counteract the cascade of inflammatory factors.

Except that, sympathetic denervation reduces sympathetic activity, changes the balance in ANS, and results in a relative increase in parasympathetic activity. This results in increased production and release of acetylcholine (ACh). Studies have demonstrated that acetylcholine regulates cytokine production by binding to nicotinic acetylcholine receptors (nAChRs) (47). A 7nAChR mainly mediates autologous/paracrine ACh to up-regulate IL-10 production (48). The binding of IL-10 to IL-10 receptor (IL-10R) leads to forms high-affinity JAKs site in the cytoplasm, which induces phosphorylation of STAT3 (49). JAKs, a family of receptor-associated cytosolic protein tyrosine kinases, rapidly transfer extracellular signals into the cell, thereby modulating gene expression (50). JAK consists of the JAK homology (JH)1 domain and the JH2 domain, and its downstream signaling molecules include STAT3, PI3K/Akt, Ras, etc. (51). The JH1 domain is responsible for the catalytic activity of JAKs (50). Previous studies have confirmed that activating the PI3K/AKT signaling pathway can reduce ROS levels in myocardial cells and inhibit cardiomyocyte autophagy in animal myocardial ischemia-reperfusion injury models, contributing to improving cardiac function (52, 53). In addition, activation of Ras signaling in cardiomyocytes is associated with the progression of pathogenic cardiac hypertrophy and subsequent heart failure (54). The activated JAKs can be combined with the Src homology 2 (SH2) domain of STAT (55). The classical signaling pathway JAK/STAT is widely recognized as an essential cardiovascular protective factor (56). Studies have demonstrated that STAT3 signal activation alleviates myocardial ischemic injury (57). However, it remains unclear whether the cardioprotective effect of sympathetic denervation in IR injured myocardium is related to the activation of the STAT3 signal.

Many of the protective effects of STAT3 can be attributed to the induction of anti-inflammatory and survival genes (58). STAT3, a key effector of IL-10, is widely considered an essential cardiovascular protective factor (58), playing a protective role in chronic myocardial remodeling after ischemic injury and immune-mediated myocarditis (59). Cardiac sympathetic activity increased within 1 h after the onset of myocardial infarction and was maintained for more than 1 week (60). IL-10 expression was first detected in animal models at 5 h after myocardial ischemia-reperfusion (61). Changes in sympathetic excitability would be preceded the production of IL-10 in the myocardial I/R model. In this study, the SCG was removed by surgery before the myocardial I/R injury, which results in local sympathetic denervation in the myocardium. Sympathetic excitability was decreased in the SCGx + I/R group compared with the I/R group, as evidenced by decreased myocardial TH level and serum NE, accompanied by elevated levels of the anti-inflammatory factor IL-10. Moreover, injury/oxidative stress would be the switch for activation of this pathway, and the SCG group is not significant for further activation of the pathway.

It is well known that increased sympathetic tone in mice with myocardial infarction occurs locally in the myocardium and throughout the body (62, 63), which is attributed to regional changes in sympathetic innervation of the heart following myocardial infarction. Studies have reported extensive denervation of the left ventricle (LV) below the infarct, while the myocardial boundary’s cardiac base shows significant hyperinnervation (64). Except for that, Neural activity stimulated the expression of the catecholamine synthesis rate-limiting enzyme TH and catecholamine production to a greater degree than NE reuptake. This may explain why myocardial infarction elevates TH to levels higher than in control animals (65). In AOGEN transgenic rats, an animal model lacking post-infarction sympathetic hyperactivity, myocardial I/R injury-induced increases in rat TH and NE transporter (NET) genes and proteins were not observed (66). This demonstrated that increased neural activity stimulates the expression of these proteins and the genes that code for them. Therefore, the changes in TH protein and NE levels can be used as indicators to measure the changes in sympathetic excitability.

Laboratory and clinical studies have proved that inhibition of systemic or local sympathetic excitability improves cardiac function in the ischemic myocardium, reducing ventricular remodeling, arrhythmias, and inflammatory responses (11, 17, 67, 68). But most of these methods are targeted at stellate ganglion. SCG plays a dominant role in the innervation of the anterior wall of the myocardium could be particularly critical for myocardial injury caused by left anterior descending branch obstruction. Unilateral or bilateral excision of part of the sympathetic chain is controversial. Previous studies have shown that both sides of SCGx contribute equally to the sympathetic innervation of the anterior wall (17, 69). In Yorkshire pigs, it has been demonstrated that the afferent signal of post-myocardial infarction transduced resulted in bilateral stellate nerve changes, and both sides of cardiac sympathetic neuron responses equally to myocardial infarction (70). In a clinical trial, bilateral sympathectomy was more beneficial than unilateral sympathectomy in patients with ventricular tachyarrhythmia (VT) storm (71). We selected bilateral superior cervical sympathectomy to construct an animal model with reduced sympathetic excitability in the heart. In our study, the SCGx + I/R group showed lower sympathetic excitability, cardiomyocyte apoptosis, and oxidative stress levels than the I/R group. Interestingly, STAT3 phosphorylation was accompanied in the SCGx + I/R group. This effect was partially attenuated by intraperitoneal administration of STAT3 phosphorylation inhibitors. We considered that the protective effect of sympathetic denervation on the myocardium is related to the activation of STAT3 signal after injury, and this effect is caused by decreased sympathetic excitability.

Our study also has several limitations. In our study, the stattic was used to specifically inhibit Tyr705 phosphorylation of STAT3, but we did not use stattic as a separate control group. Many studies have shown that the toxicity of stattic is related to its plasma drug concentration. 100 μM stattic treatment for induced STAT3 and tubulin degradation confirming that high stattic concentration could trigger toxic effects independent of STAT3 action (72). However, *in vivo* experiments of mice, studies have shown that low concentrations of stattic did not cause additional toxicity (73). Similarly, Das et al. have demonstrated that stattic with 20mg/kg (i.p.) did not affect cardiac shortening fractions in normal mice (74).

Signal transducer and activator of transcription 3 transcriptional activity is regulated by phosphorylation of two separated residues. But in our study, we only explored the Tyr705 phosphorylation site. STAT3 has 14 highly conserved cysteine residues, nine of which are reported to be sensitive to redox activity and are closely related to the activation of tyrosine residue phosphorylation (Tyr705) (20–22). On the other hand, the function of Ser727 phosphorylation remains controversial, phosphorylation of Ser727 has been reported to have both activating and inhibitory effects on STAT3 transcriptional activity (75, 76). More recently, it has been demonstrated that phosphorylation of Tyr705 is absolutely required for STAT3-mediated ESCs self-renewal, whereas phosphorylation of Ser727 is dispensable, serving mainly to promote proliferation and multi-directional differentiation (77).

The currently available data indicate that a complex network of signaling mechanisms is involved in STAT3 regulation, involves both genomic effects and mitochondrial effects, much more await exploration. It is necessary to discuss further the relationship between the change of TH level and protective myocardium injury after decreased sympathetic excitability.

## Conclusion

**FIGURE 7:**
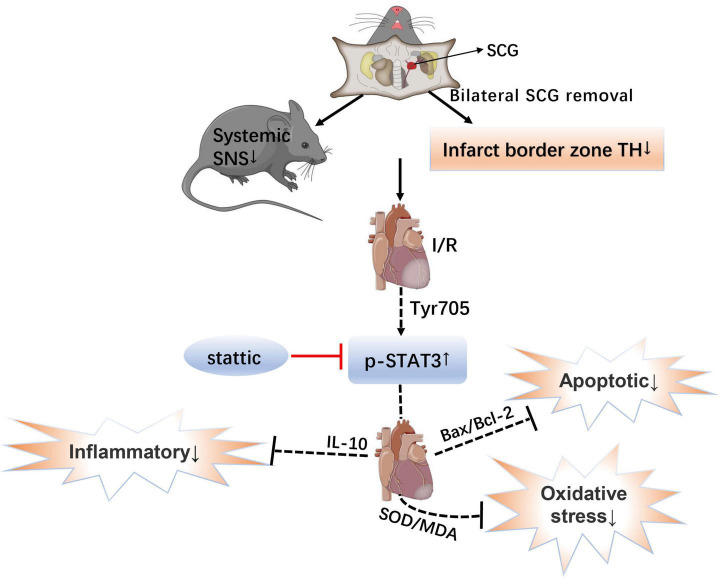
Schematic showing the mechanistic role of the STAT3 signal in myocardial ischemia-reperfusion injury after bilateral superior cervical sympathectomy. SCG, superior cervical ganglion; SNS, sympathetic nervous system; I/R, ischemia-reperfusion; TH, tyrosine hydroxylase; p-STAT3, The transcription factor, signal transducer and activator of transcription 3; IL-10, Interleukin 10; SOD/MDA, superoxide dismutase/malondialdehyde; and Bax/Bcl-2, Bcl-2 Associated X protein/B-cell lymphoma 2.

These data favor the notion that bilateral superior cervical sympathectomy may be considered a new therapeutic strategy for treating myocardial I/R injury. The present study provides evidence that removal of SCG pretreatment may contribute to the protection of myocardium against I/R injury by activation of STAT3 signaling *via* down-regulating sympathetic excitability, which is achieved by reducing the level of oxidative stress, alleviating apoptosis and decreasing the level of inflammatory reaction (Figure 7). This study sheds new light on the molecular mechanisms whereby sympathetic denervation may ameliorate the myocardial I/R injury.

## Data Availability Statement

The raw data supporting the conclusions of this article will be made available by the authors, without undue reservation.

## Ethics Statement

The animal study was reviewed and approved by the Institutional Animal Care and Use Committee of Zhujiang Hospital of Southern Medical University.

## Author Contributions

LiL contributed to the data curation, statistical analysis, and writing of the original draft. JG and LG participated in data collection. LeL and HZ participated in the study design. WZ and SX conceived and designed the experiments. All authors contributed to the article and approved the submitted version.

## Conflict of Interest

The authors declare that the research was conducted in the absence of any commercial or financial relationships that could be construed as a potential conflict of interest.

## Publisher’s Note

All claims expressed in this article are solely those of the authors and do not necessarily represent those of their affiliated organizations, or those of the publisher, the editors and the reviewers. Any product that may be evaluated in this article, or claim that may be made by its manufacturer, is not guaranteed or endorsed by the publisher.
