# Supplementary material for: Bilateral Superior Cervical Sympathectomy Activates Signal Transducer and Activator of Transcription 3 Signal to Alleviate Myocardial Ischemia-Reperfusion Injury
Source: Front Cardiovasc Med. 2022 Apr 1;9:807298. doi: 10.3389/fcvm.2022.807298 (PMC9010611; doi:10.3389/fcvm.2022.807298)
Supplement: Supplementary file 1 [file Data_Sheet_1.pdf]

Supplemental material

Figures and Legends

Figure S1

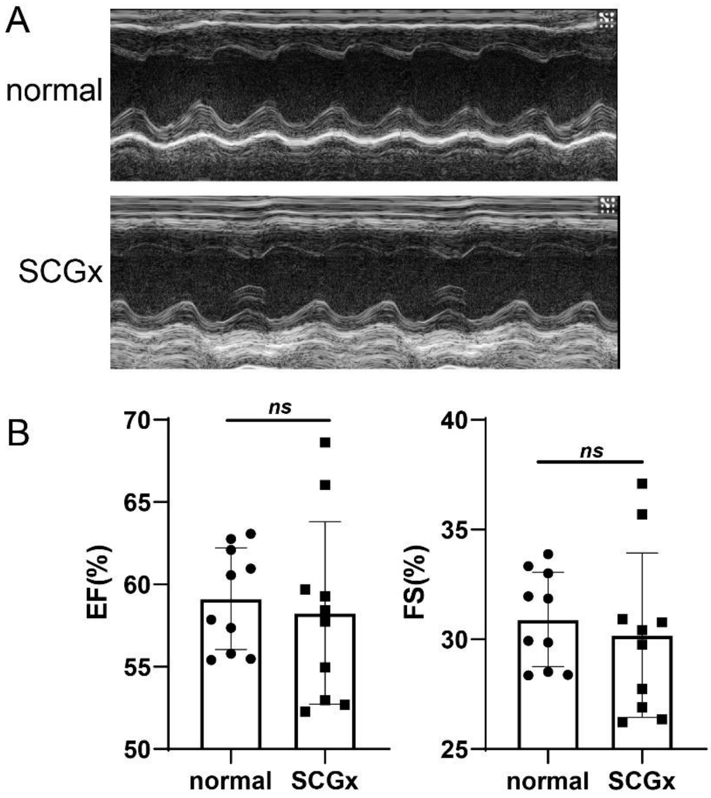

Figure S1. Cardiac function was evaluated by echocardiography after Bilateral superior cervical sympathectomy. (A) Representative M-mode echocardiography images. (B) LVEF, left ventricular ejection fraction. LVFS, left ventricular fractional shortening. (n=10), *ns*, None significance.

Figure S2

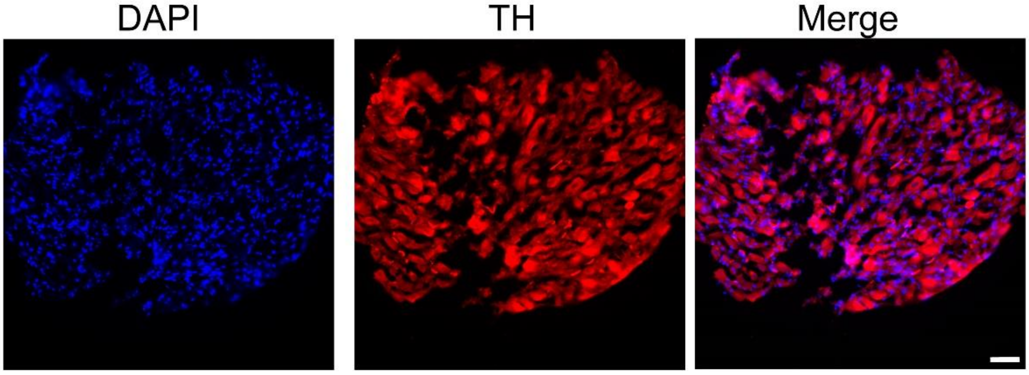

Figure S2. Immunofluorescent staining of surgically removed SCG. scale bar: 50μm.
